# Exploring the structure of the school curriculum with graph neural networks

**DOI:** 10.1007/s42001-025-00420-9

**Published:** 2025-09-03

**Authors:** Benjamín Garzón, Vincenzo Perri, Lisi Qarkaxhija, Ingo Scholtes, Martin J. Tomasik

**Affiliations:** 1 Chair of Research Methods in Developmental and Educational Sciences, Institute of Education, University of Zurich, Zurich, Switzerland; 2 Chair of Machine Learning for Complex Networks, Center for Artificial Intelligence and Data Science, Julius-Maximilians-Universität Würzburg, Würzburg, Germany

**Keywords:** School curriculum, Computer-based assessment, Graph neural networks, Educational measurement, Online assessment, Machine learning

## Abstract

School curricula guide the daily learning activities of millions of students. They embody the understanding of the education experts who designed them of how to organize the knowledge that students should acquire in a way that is optimal for learning. This can be viewed as a learning ’theory’ which is, nevertheless, rarely put to the test. Here, we model a data set obtained from a Computer-Based Formative Assessment system used by thousands of students. The student-item response matrix is highly sparse and admits a natural representation as a bipartite graph, in which nodes stand for students or items and an edge between a student and an item represents a response of the student to that item. To predict unobserved edge labels (correct/incorrect responses) we resort to a graph neural network (GNN), a machine learning method for graph-structured data. Nodes and edges are represented as multidimensional embeddings. After fitting the model, the learned item embeddings reflect properties of the curriculum, such as item difficulty and the structure of school subject domains and competences. Simulations show that the GNN is particularly advantageous over a classical model when group patterns are present in the connections between students and items, such that students from a particular group have a higher probability of successfully answering items from a specific set. In sum, important aspects of the structure of the school curriculum are reflected in response patterns from educational assessments and can be partially retrieved by our graph-based neural model.

## Introduction

### The school curriculum and the structure of school knowledge

Millions of students worldwide are learning every day according to their respective school curriculum. The curriculum provides a structured framework designed to guide the sequence of teaching activities that should form the educational process, reflecting at the same time the body of knowledge that a society finds relevant and what is considered the optimal learning progression. Although organized education has existed since ancient civilizations, the modern Western conception of curriculum was developed in the 19th and 20th centuries by educational thinkers such as Lester Frank Ward, John Dewey, and Horace Mann, and continues to evolve today [1].

To be useful for the development of informative educational assessments and effective learning methods allowing to build a solid foundation in the different school subjects, the curriculum should capture the relationships between the concepts and competences that students are supposed to acquire. From a philosophy of science perspective, the curriculum can therefore be regarded as a theory that describes the relationships between the pieces of knowledge contained in the different school subjects. In spite of its utmost relevance for guiding instruction and learning, this theory outlined by the curriculum is nevertheless hardly scrutinized empirically. This differs markedly from other socially relevant areas where scientific approaches have been extremely successful, such as health care, for which evidence-based practice and rigorous testing are standard [2, 3]. Reasons for this apparent discrepancy may be that curricula are shaped by a combination of historical context, societal needs, ideological and political influences, and technological advancement; that educational theories are vague and difficult to formalize; that collecting the data for rigorous and comprehensive empirical testing was a challenge until recent years; and that analytical tools for such task are lacking.

When assessments are designed in accordance with the curriculum they provide an opportunity to test if the actual performance patterns of students are in agreement with the assumptions embodied in the curriculum. The understanding that educational experts have of the relationships between pieces of knowledge is reflected in the way they assign assessment items to the different classes of a taxonomy that may span several hierarchical levels (e.g., subject domains, competence domains, single competences). For example, an item asking for the solution to a particular arithmetic operation can be assigned to the subject domain of mathematics, the competence domain "numbers and variables", and the competence described as "the students can represent, exchange and understand calculation methods for basic operations with decimal numbers." However, any subjective categorization is susceptible to inaccuracy and bias, and answering certain items may require multiple competences, making it difficult to assign them to a single category. It is impractical for human experts to objectively evaluate the myriad relationships between all items that students encounter during the educational process. Fortunately, the advent of digital technologies in educational practice has provided access to large and rich data sets collected in more ecological conditions than standardized assessments that can be used to inform educational decisions and ultimately benefit student achievement outcomes [4, 5]. These observations constitute a more principled alternative to the informal interactions between teachers and students through which the former attempt to form an opinion on the capacities of the latter, often relying on intuition and partial observations from heterogeneous situations [6, 7].

Curriculum development is an arduous undertaking that encompasses a multitude of different aspects [8]. To ensure that they promote learning in the best possible way, curricula should ideally be designed primarily on the basis of direct research evidence rather than on subjective assumptions. For this reason, data-driven approaches to curriculum design are becoming more common [9–11]. Reliance on automated curriculum design methods can favour an iterative process enabling more frequent updates of optimized school curricula. An accurate and objective categorization of the items can help to avoid making modifications to the curriculum that do not meet the needs of the students (and can result in a loss of precious resources [12]), ultimately leading to better recommendations of items and learning paths.

### Modeling data from computer-based assessment systems

Computer-based formative assessment (CBFA) systems are software tools designed for data collection and performance evaluation in the classroom with the aim of providing feedback and supporting instructional decisions. CBFA systems enable the acquisition of large-scale data sets that can be used to study academic abilities and their development in everyday settings, in conditions with higher ecological validity compared to those found in standardized assessments. In the present study, we modeled a data set with more than 30 million assessment responses obtained from a CBFA system serving a population of tens of thousands of students in Northwestern Switzerland and spanning four school subject domains: mathematics, German (as the language of instruction), as well as English and French (as the two foreign languages taught). The students responded to the items either as part of regular school activities or at home. The system includes an item bank containing thousands of items that cover topics and competences over several years of mandatory schooling, from grade 3^1^ to grade 9. The items were designed according to Lehrplan 21, the competence-based curriculum currently used in the German-speaking cantons in Switzerland [13]. The items of each subject domain are further categorized in competence domains: ’numbers and variables’, ’form and space’ and ’measures, functions and probability’ for mathematics; ’reading comprehension’ and ’grammar’ for German; and ’reading comprehension’, ’listening comprehension’ and ’grammar’ for French and English, giving a total of eleven competence domains. The competence domains are further subcategorized in different competences.

A difficulty that arises when collecting data under the more unconstrained conditions enabled by such a system is that a given student will only answer a small subset of the items available in the bank, and this subset will differ between students. The resulting student-item response matrix is thus large and highly sparse, and not naturally suited for common test-scoring techniques like Item Response Theory (IRT; Reise et al.; Carlson; Embretson and Reise [14–16]). These classical methods were developed mainly for small data sets with few missing data and have low expressiveness, thwarting the possibility of extracting valuable information from large-scale data sets that are becoming increasingly available thanks to the proliferation of digital technologies. To tackle these challenges, we resorted to Graph Neural Networks (GNNs; Hamilton [17]), a class of machine learning methods recently developed to model graph-structured data.

### Graph neural networks

**Fig. 1:**
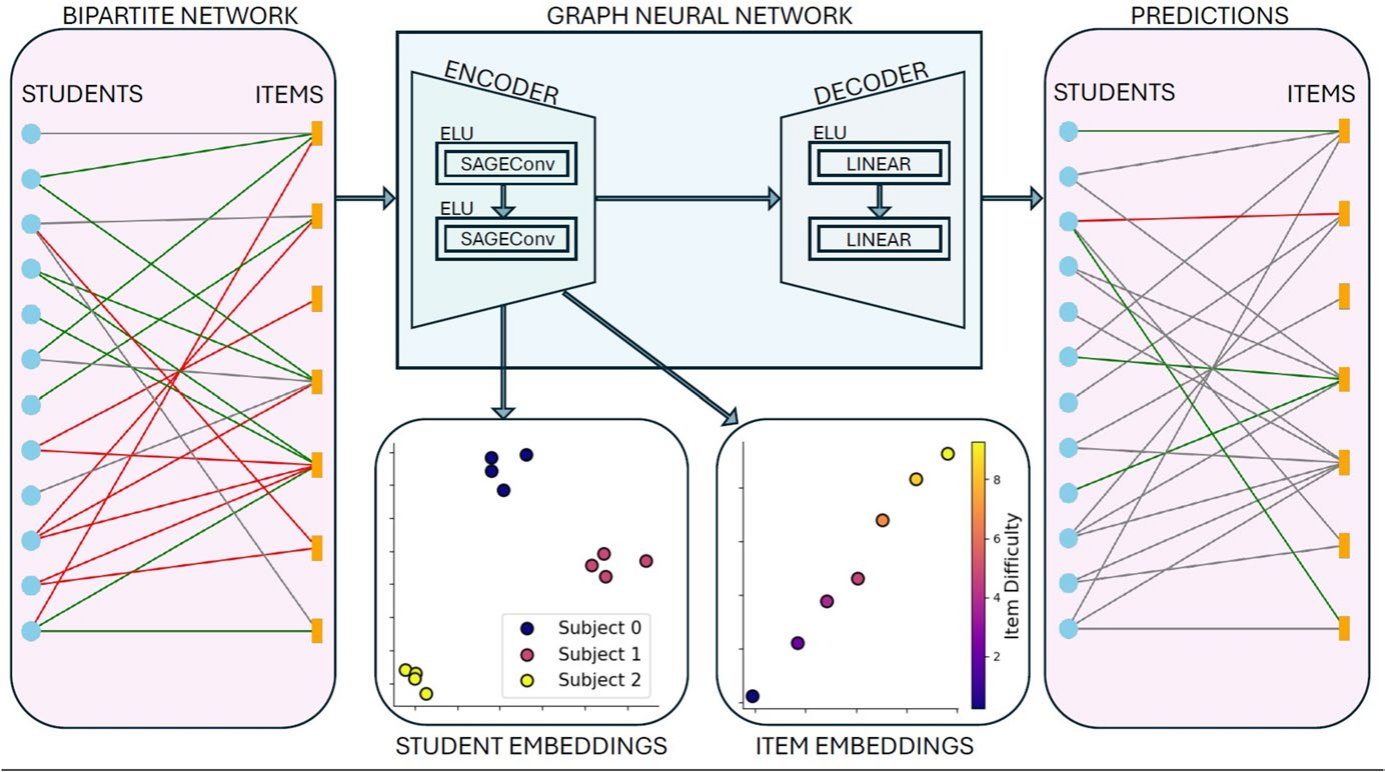
Analysis pipeline. The data used as input for the analysis is represented as a bipartite graph where interactions between nodes in the separate partitions correspond students responding to items. The data set is highly sparse, as a given student answers only a small fraction of all possible items. We analyze the data with a Graph Neural Network (GNN) because of its ability to include students and item features and account for the graph of student-item interactions. The output of the analysis is shown on the right. From the model, we obtain both predictions for interactions (correct or incorrect) and vector representations (embeddings) for the students and items. Thanks to the GNN’s ability to combine features and topology, these data-driven representations enable us to investigate relationships between items in the school curriculum

A *graph* is a flexible data structure consisting of nodes or vertices, which represent entities of interest, and edges, representing the relationships between those entities, which can be of a diverse nature. In this work, we consider a *bipartite* graph (i.e., with edges connecting two different types of nodes) where the nodes can refer to students or items, and the edges link student-item pairs, denoting that the student has responded to the item. GNNs learn multidimensional node vector representations called *embeddings* that depend on both the structure of the graph and the features of the nodes (e.g., student gender), so that nodes with similar characteristics are represented with similar embeddings [17]. A common application of GNN models is in supervised learning tasks, where the goal is to predict given labels of either nodes or edges. The latter is relevant for the current scenario, where our aim was to predict whether a student answered an item correctly or not (i.e., the edge label: correct/incorrect) in the aforementioned data set of online school assessments (Fig. 1).

In our work, we used the node and item embeddings learned by the GNN to empirically validate the school curriculum. Specifically, we compared the concept relations posited by curriculum designers and the proximity between pairs of these item embeddings. This analysis evaluated the alignment between the assumed concept relationships reflected in the curriculum structure and the patterns in the actual response data of students, as captured by the GNN embeddings. Thereby, we could assess the validity of the curriculum’s underlying assumptions about how academic concepts relate to each other based on real-world evidence.

## Results

### Model performance

As outlined above, we train the GNN model for a binary classification task, where the model predicts the correct/incorrect label for each edge representing the answer of a student to an item. Upon convergence of the GNN model, the test area under the receiver operating characteristic curve (AUC) was 0.82 and the balanced accuracy 0.74.

### Interpreting the embeddings

**Fig. 2:**
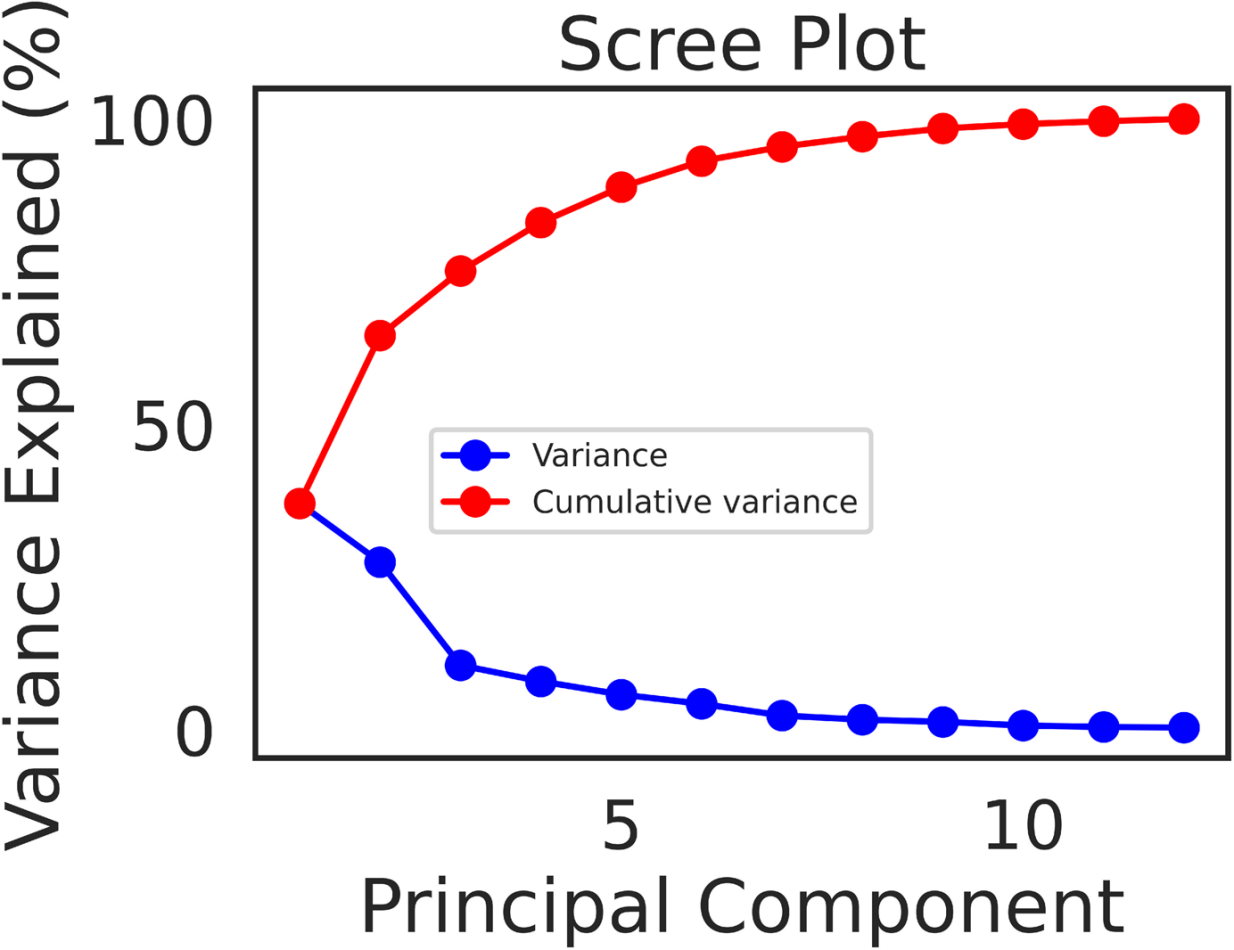
*Variance decomposition.* Scree plot of the Principal Component Analysis of the item embeddings

**Fig. 3:**
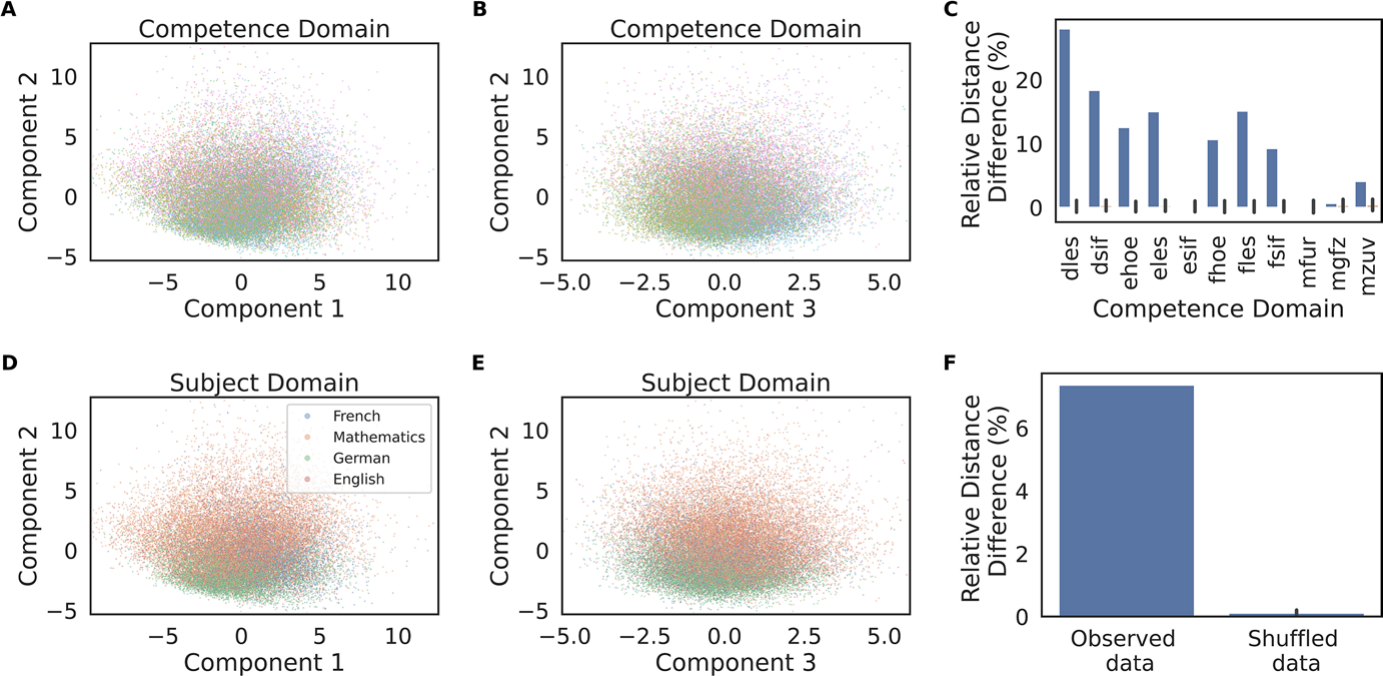
Item embeddings and subject/competence domains. **A** Second v. first components and **B** Second v. third components of the Principal Component Analysis (PCA), with instances colored by *competence domain* (i.e., German Grammar, German Reading, French Grammar, ...), showing that the model learned similar embeddings for items of the same competence domain. The color labels are omitted for clarity, as there were 11 different competence domains. **C** Accordingly, the Euclidean distance between learned item embeddings of different competence domains was larger than between embeddings of the same domain (blue bars). This was not the case when the competence domain labels were shuffled (indicated by the bars with black error lines representing the standard deviation; note that the bars show the relative distance difference, which should be zero on average for the shuffled data, making the bars barely visible). **D** Second v. first components of the PCA and second v. third components **E**, with instances labeled by *subject domain* (i.e., German/French/English/Mathematics), showing clustering within these categories. **F** Accordingly, when considering pairs of learned item embeddings of *different* competence domains, the Euclidean distance was larger when the items belonged to different subject domains than when they belonged to the same subject domain. Therefore, the model learned to represented items from the same subject domain with similar embeddings

The scree plot of the Principal Component Analysis (PCA) of the learned item embeddings and parallel analysis [18] indicated that there were 3 significant components which explained more than 75 % of the variance in these embeddings (Fig. 2), with the first component explaining 37 % of it. In order to interpret these embeddings, we examined the scores corresponding to the components from this low-dimensional PCA projection. Item embeddings of the same competence domain (i.e., German Grammar, German Reading, French Grammar, ...) tended to cluster together (Fig. 3A, B), such that the distance between two items was on average up to 28 % larger when they belonged to different competence domains than when they belonged to the same (Fig. 3C), and for most competence domains this difference was significantly above what would be expected by chance (the bars in Fig. 3C show a *relative distance difference*, which should be zero on average for the shuffled data). Item embeddings of the same subject domain (i.e., German/French/English/Mathematics) also tended to cluster together (Fig. 3D, E), such that the distance between two items was on average about 8 % larger when the items belonged to different subject domains than when they belonged to the same (Fig. 3F). Note that while we entered the competence domain as a feature in the model, the model was agnostic about the subject domain but it still learned to place items of the same subject domain closer together to increase predictive accuracy.

**Fig. 4:**
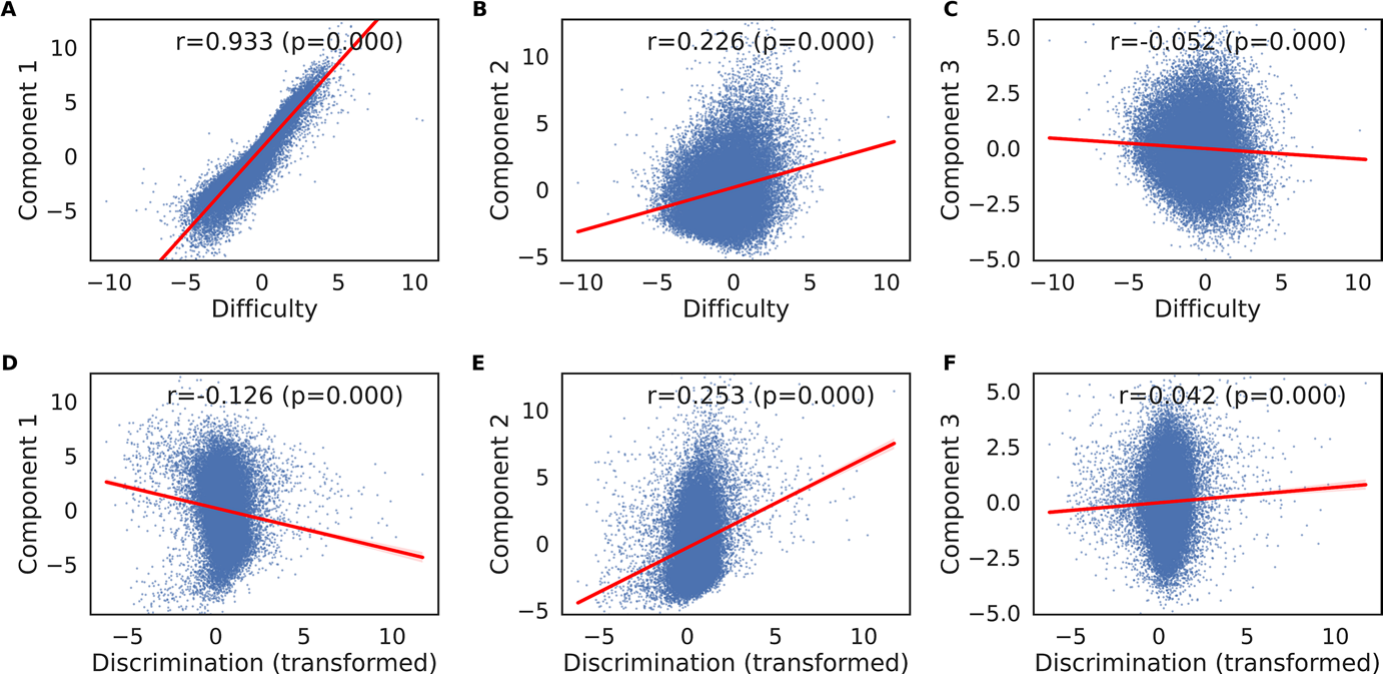
Item embeddings against IRT parameters. Upper row: scores of the first **A**, Second **B**, and third **C** Components of the item embeddings against the difficulty parameter obtained with the IRT model. Lower row: scores of the first **D**, Second **E**, and third **F** Components of the item embeddings against the discrimination parameter obtained with an IRT model

We also investigated relationships of the item embeddings with item parameters as assessed by an IRT model, namely difficulty and discrimination (see Appendix C for a definition of these parameters). The scores of the first component of the item embeddings was strongly associated with IRT item difficulty (Fig. 4A), while IRT item discrimination was most strongly associated with the scores of the second component (Fig. 4E). In other words, the GNN model captured these interpretable dimensions estimated by the IRT model, and most of the variance in item embeddings (accounted for by the first component) was related to their difficulty, as expected.

**Fig. 5:**
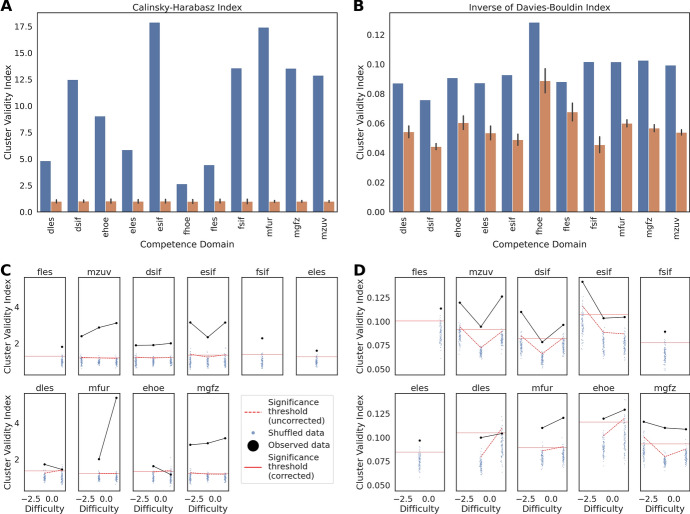
Clustering of item embeddings. **A** Calinski-Harabasz cluster validity index obtained for each competence domain when labeling the embeddings with expert-defined competences. The orange bars denote the index values after permuting the competence labels. The cluster validity indices were significant for all the competence domains. **B** The same as in **A** for the inverted Davies-Bouldin cluster validity index. **C** Analysis of cluster validity indices when subdividing the items for each competence domain by difficulty level. The blue dots indicate the cluster validity index values obtained after permutation of the competence labels, the dashed red lines the value of the index corresponding to a p-value $p=0.05$ (considering the null distribution obtained by permutation of the competence labels), and the continuous red line the value of the index that corresponds to the significance after correction for multiple comparisons ($p=0.05$, considering the null distribution obtained by permuting the competence labels and taking the maximum across difficulty levels). This analysis focused on the difficulty levels with enough observations to enable robust statistical comparisons (see Methods; for the competence domains not shown there was insufficient data across all difficulty levels). **D)** Panel equivalent to **C)**, but for the inverted Davies-Bouldin cluster validity index

Finally, we asked whether the structure of the item embeddings corresponded to the structure of competences in the curriculum as defined by human experts. For each competence domain, we computed cluster validity indices with respect to the labels defined by the different competences to ascertain whether items of the same competence had on average more similar embeddings than it would be expected by chance. This was indeed the case for all competence domains (Fig. 5A, B; see Methods for details). Note that no information about the identity of these competences was introduced as a feature in the model. We also verified that the clustering of the embeddings was not only reflecting the fact that items corresponding to different competences had different levels of difficulty. When dividing the items further by difficulty level within each competence domain, the cluster validity indices obtained for some of these subdivisions were still well above what would be expected by chance (Fig. 5C, D). These effects were more marked for the Calinski-Harabasz (Fig. 5A, C) than for the Inverted Davies-Bouldin index (Fig. 5B, D). All in all, these results indicate that the model was able to learn important aspects of the structure of the academic curriculum. That is, encoding items with similar embeddings when they had similar curricular attributes was advantageous in order to predict responses.

## Discussion

The school curriculum acts as a central source of guidance in educational practice. The development of curricula is influenced by many factors, such as pedagogical research, educational theory, practical considerations, political views, and social attitudes. To maximize learning effectiveness, curriculum development should ideally rely on direct research evidence rather than subjective opinions of a limited number of experts. The emphasis on evidence-based practice in education has grown over the past few decades [6, 19, 20], and more recently this trend has also included data-driven curriculum development [10, 11, 21].

In this study, we modeled millions of student responses to thousands of items from an online formative assessment over the course of mandatory education to test how the curriculum structure translated into the structure of students’ performance. The data set we analyzed in the present work, spanning the whole period of mandatory schooling, is exceptional in its magnitude and scope, which allowed us to examine the curriculum structure comprehensively and the myriad relationships between items at once, instead of focusing only on a narrow part of it. To deal with the large-scale, sparse structure of these real-world data, we developed a GNN model based on a bipartite graph with separate embedding representations for students and items.

The learned item embeddings clustered according to subject and competence domains. The distance between items of different subject/competence domains was on average greater than between items of the same domain. This difference was generally significant, although small (relative increase of less than 30%), resulting in clusters that were not clearly defined. This is not surprising given that academic skills are known to be strongly correlated [22, 23]. Most of the variance in item embeddings was accounted for by only 3 components. Unsurprisingly, the first principal component, accounting for most of the variance in item embeddings, was highly correlated with item difficulty as assessed by a standard IRT model. The item embeddings were also associated with item discrimination, most notably for the second PCA component, presenting thus a differential pattern of correlation with these components compared to the difficulty parameter. Overall, this indicates that these two parameters were important sources of differences between item embeddings. Our analyses also showed that the learned item embeddings reflect both subject and competence domains and to some extent recapitulate the structure of the competences defined by the experts, even when controlling for difficulty level. Even the (inverted) Davies-Bouldin index, a stringent measure that compares the distances within each cluster with the distance to its most similar cluster, was above chance for some of the difficulty-binned subsets, indicating that (with enough and adequate data) the learned embeddings reflect well the competences for those subsets. Together, these observations highlight the ability of the model to capture finer-grained information from response data. Future work should further investigate the data quality requirements (e.g., reliability level) needed to achieve a satisfactory retrieval of competences.

Although other techniques such as multidimensional IRT (MIRT; Reckase [24]) also employ a multidimensional representation of item parameters, our approach is more expressive in that the function relating item and student parameters, which is learned from the data, is more flexible and can capture complex item dependency patterns through its neural message passing mechanism. This can be an advantage in large-scale scenarios where imposing a rigid structure may lead to underfitting. Although in our empirical data set the GNN did not outperform an IRT model, experiments on synthetic data (Appendix D) demonstrated that the GNN exhibited a performance advantage when group patterns were present in the connections between students and items, such that students from a particular group had a higher probability of successfully answering items from a specific set (e.g., topic), in which case the GNN can leverage topological information to improve prediction. These group patterns may arise if students are tested more frequently on items they know well, as when the assessment is part of the learning process (which was not the case here). Similar patterns may also emerge under the influence of factors such as shared student backgrounds, common instructional approaches within student groups, or student preferences, which are plausible occurrences in real-world educational settings. For instance, the school-level intra-class correlation coefficients for math and reading achievement can exceed 0.2, which implies that students of the same school tend to perform similarly to each other to some degree [25].

Our approach has the potential for practical application in identifying curriculum design issues in several ways, which can be valuable to inform curriculum development: 1) Detecting items that are very similar (in terms of their psychometric properties), so that those that are redundant can be filtered out, or new items can be generated by combining them. Since learners have limited time and energy, it is desirable to design efficient learning materials that avoid unnecessary effort. A similar scenario arises in the case of computerized adaptive testing and optimal test construction [26, 27], which aim to reduce the burden on test-takers by selecting the most informative items so that an assessment can be made with a minimal set of items. 2) Identifying parts of the syllabus with a lower density of items, so that these parts can be conveniently expanded. 3) Recognizing items that are thought to reflect a particular category (e.g., competence, topic), but which differ significantly from items in the same category from a performance point of view, suggesting that they should be revised. 4) Identifying the most important item features for performance prediction (in cases where, unlike here, multiple such features are available), which provides useful information for designing new items. Post-hoc interpretability methods [28] can be used to pinpoint relevant features in complex architectures. 5) Finding optimal ways to traverse the item embedding space in order to recommend optimal learning paths. Learning path recommender modeling [29–31], is an active area of research that aims to design systems that personalize sequences of learning materials based on the learner’s individual goals and needs, thereby enhancing their experience and attainment. This task can be accomplished with a comprehensive parametric representation of the items, such as the multidimensional embeddings obtained by our framework. Learning path models often rely on graph-based data representations [32, 33] where nodes represent items and edges represent relationships between them.

In addition to learning path models, there are other examples of methodological perspectives that share the core assumption that learning is shaped by the organization of knowledge elements and their interrelations. Knowledge space theory [34] is a theoretical framework that uses mathematical formalisms to represent knowledge structures within a given domain. It formalizes dependencies between items, such as which items are prerequisites for a particular item, using graphs. Another example is knowledge tracing, a technique used in intelligent tutoring systems to model the level of mastery of specific skills (knowledge components) attained by learners [35, 36] as they interact with learning materials over time. Recently proposed methods in this field resort to graph-based deep learning techniques to model the relationships among items and knowledge components [37, 38]. In essence, graph-based approaches are ideally positioned to harness the abundant relational structures found in educational data. Combined with the increasing volume of educational data, this points to promising directions for GNNs in this field.

Our work has several limitations that should be noted. One downside of our approach compared to more parsimonious and explicit models is that the item embeddings do not have a direct interpretation, although this was not an essential feature for the purpose of understanding the between-item correlations in performance that we were concerned with here. GNNs may nevertheless offer better interpretability than standard feed-forward neural networks for tasks involving relational data because of their ability to model complex relationships through their graph structure and message passing mechanism. This allows for a more intuitive understanding of how information flows through the graph and it has been used in explainable methods to identify which subgraphs are important for prediction [39]. GNNs have also been shown to act as concept detectors, exhibiting strong alignment with concepts formulated as logical compositions of node degree and neighborhood properties [40].

The high degree of sparsity of the sample, with large differences between students in the number of items they responded to, means that the reliability of learned item embeddings may vary widely across items. A further limitation of our model is that it does not account for these differences in reliability; taking them into account is likely to be important in accurately categorizing items into competences, topics, etc. Furthermore, the selection of items with which students interacted reflected a mix of teacher choice and system recommendations, but was not optimized for prediction. A more sophisticated recommendation scheme could lead to less noisy estimates. As another strategy for reducing uncertainty, future efforts could attempt to homogenize testing conditions by controlling certain factors of a session if this does not entail increasing the burden on students (e.g., by testing at regular times or by checking that the environment meets certain requirements). An alternative would be to collect detailed information about testing conditions (e.g., by asking test takers to report whether a test session took place at home or at school) or other factors that may influence performance, which could be entered as model features. In general, high levels of noise in the response data may have dampened the estimation of performance correlations, preventing the full benefits of the GNN model from being realized.

## Conclusion

Understanding the effect of the curricular structure on students’ performance is an important step toward validating the curricular design, paving the way for implementing future improvements. Using a large-scale data set of online educational assessments with millions of responses, we show that key elements of the school curriculum structure can be partially recovered from the students’ response patterns.

## Methods

### Formative assessment data set

The CBFA system MINDSTEPS (https://www.mindsteps.ch/) has been used in Northwestern Switzerland over several years to collect objective information about students’ current abilities and learning progress for four school subject domains: mathematics, German (the official language in the region where the schools are located), English and French (the two foreign languages taught). The MINDSTEPS CBFA system serves a population of thousands of students in four German-speaking cantons of Switzerland and consequently has a substantial impact on digital learning both within and outside the school environment. The system automatically evaluates students’ responses, with wrong or omitted responses scored as 0, and correct responses scored as 1. A more comprehensive overview of the data and the development of the CBFA can be found in [41] and further details on the items and their administration in [42].

#### Ethics approval statement

All procedures performed in studies involving human participants were in accordance with the ethical standards of the institutional research committee and with the 1964 Helsinki Declaration and its later amendments or comparable ethical standards. In line with the American Psychological Association’s Ethical Principles and Code of Conduct, as well as with the Swiss Psychological Society’s Ethical Guidelines, written informed consent from the students and their parents was not required because this study was based on the assessment of normal educational practices and curricula in educational settings. The Institute for Educational Evaluation as a contractor of the cantonal educational authorities committed to obeying the laws of the four cantons involved to ensure strict data confidentiality. In line with the laws of the four cantons, approval from an ethics committee was not required for this study.

### Data preparation

CBFA responses were discarded if they corresponded to unfinished assessment sessions. Students included in the analyses were limited to those attending public schools and for whom age, grade, gender, and mother tongue information was available. To ensure the reliability of the model parameters, we excluded items with a probability of correct responses greater than 90 % or less than 10 % (since these items provide minimal information). Participants who had answered fewer than 30 questions and questions with fewer than 30 responses were likewise excluded. We considered these decisions to be sensible and implemented them before conducting any further analyses. After these preparatory steps, the final data set used for the subsequent analyses comprised 91,677 students, 35,087 items and 33,767,508 responses, making its size and scope rare for a data set in this domain.

### Learning embeddings of assessment items

Our goal is to learn a vector representation of the items of the school curriculum using CBFA data; the peculiarity of these data sets is that they are big but sparse, given that each student interacts with only a few items. Traditional psychometric methods such as Item Response Theory (IRT; Reise et al.; Carlson; Embretson and Reise [14–16]) were designed for smaller data sets with denser observations and lack the expressiveness of deep learning techniques. In contrast, GNNs excel at modeling graph-structured data sets containing numerous, often sparse, interacting components, and can learn representations that reflect both the features describing individual data instances (such as students’ mother tongue and age) and the structural graph information (i.e., interaction of students with specific items).

#### Bipartite graphs

A graph is mathematically defined as an ordered pair $G=\left( V, E\right)$, where *V* represents the set of nodes and $E\subseteq V \times V$ denotes the set of edges. In the present context, the nodes correspond to students and items, respectively, and the edges denote a student responding to an item. These interactions may be repeated, allowing multiple edges between a given student-item pair. Therefore, the set of nodes *V* is divided into two distinct subsets $V_s$ and $V_i$: $V_s$ for students and $V_i$ for items. This division, where $V=V_s \cup V_i$ and $V_s \cap V_i = \emptyset$, makes the graph *bipartite*. Furthermore, edges are described as *labeled* because the interaction of a student with an item is associated with a label $l \in \{0,1\}$ which depends on whether the student successfully answers the item ($l=1$) or not ($l=0$). Lastly, the nodes and edges have individual attributes. Students are associated with features $f_s \in \mathbb {R}^{d_s}$, while items are associated with features $f_i \in \mathbb {R}^{d_i}$. Edges can also have corresponding features $f_e \in \mathbb {R}^{d_e}$ (e.g., the age at which the item was responded).

#### Graph neural networks

A Graph Neural Network (GNN) is a type of neural network designed for modeling graph data. The core idea behind GNN models is to generate node vector representations (embeddings) that depend on the structure of the graph and on any feature information that might be available. This is achieved through a mechanism known as *neural message passing*. Through this mechanism, node representations (also referred to as *messages*) are iteratively aggregated from neighboring nodes and updated using standard feed-forward neural networks. The number of iterations of this process is determined by the number of *layers* of the model. In its basic form [43, 44], the computations for the $k^{th}$ iteration (layer) are described by the following formula:

1 $$\begin{aligned} h_u^k = \sigma \left( \textbf{W}_{self}^{k} h_u^{k-1} + \textbf{W}_{neigh}^{k} \sum _{v \in \mathcal {N}(u)} h_v^{k-1} + b^{k} \right) \end{aligned}$$

where $h_u^k \in \textbf{R}^{d^k}$ denotes the vector representation of node *u*, of dimensionality $d^k$; $\textbf{W}_{self}^{k}$ and $\textbf{W}_{neigh}^{k} \in \mathbb {R}^{d^k \times d^{k-1}}$ are trainable parameter matrices that encode the node representation update from the node’s own representation and those of its neighbors, respectively; $\mathcal {N}(u)$ indicates the neighbors of node *u*; $b_k \in \textbf{R}^{d^k}$ is a bias term; $\sigma$ is a non-linear activation function. The resulting node representations can then be used for general machine learning tasks, whose performance is evaluated through a *loss function*. Then, the gradient of the loss function is used to update the parameters $\varvec{W}^k$ of all layers *k* of the GNN. Notice that it is this process that refines the node representations, moving them towards values that enhance the model’s performance on the designated machine learning task.

In this study, we use a GNN to address a binary classification task related to edge labels. Specifically, our goal is to predict whether the students respond to the items correctly. Notice that, unlike the description provided in the previous paragraph, our data set encodes information on relatedness between nodes in a bipartite network. Nevertheless, the application of a GNN, originally designed for homogeneous graphs, can be extended to heterogeneous ones with relative ease. To address this, we adopt the methodology proposed in Schlichtkrull et al. [45] and introduce distinct relation-specific transformations based on the type and direction of each edge, i.e., whether it connects an item to a student or a student to an item. This involves considering two distinct matrices of parameters, $\textbf{W}^k_{i\rightarrow s} \in \mathbb {R}^{d^s \times d^i}$ and $\textbf{W}^k_{s\rightarrow i} \in \mathbb {R}^{d^i \times d^s}$, which represent the transformation of aggregated features from the other set partition. In the context of our work, these parameter matrices encode how student representations are updated based on neighboring items, and how items representations are updated based on neighboring students. The matrix $\textbf{W}_{self}^{k}$ retains its role of allowing the representation $h_u^k$ at layer k to incorporate information from the previous layer’s representation $h_u^{k-1}$ for the same node. Intuitively, this role is preserved by adding self-connections to each node in the data. Details about the model architecture and the experimental setup are provided in Appendices A and B.

### Interpreting the embeddings

The items that compose the assessments the students completed were designed by human experts to reflect curricular competences. In this context, a competence can be viewed as a group of items. This categorization typically takes the form of a hard classification: an item belongs to one competence, which reflects the fragment of knowledge required to respond correctly. These competences can in turn be grouped into larger units, such as competence domains (e.g., German grammar), in a nested hierarchy which may span several levels. For example, suppose an item belongs to the competence ”understanding calculation methods for basic operations with decimal numbers” within the “variables” competence domain, within the subject domain of mathematics. This labeling in a single competence does not capture the notion that answering the item correctly is likely to require an understanding of other competences, even those belonging to a very different subject domain (a good command of language grammar will be important to answer complex mathematics items). A better characterization of the items could be obtained by placing them in an abstract space in which the distance between different items represents their similarity in terms of performance. In other words, two items with correlated performance across examinees should lie close by in this space, reflecting that they require similar competences. Machine learning algorithms can leverage the correlations in performance to learn these items representations and use them for prediction purposes. Given the learned representations, items can then be easily classified in soft categories, tantamount to competences defined on the basis of performance.

First, we performed a PCA of the item embeddings to evaluate the number of relevant dimensions and examined their low-dimensional projections. We estimated the number of components with parallel analysis [18]. To ground these components in interpretable parameters, we fitted an IRT model to the response data and estimated the difficulty *d* and the discrimination *a* parameters for each item *j*. Subsequently, we correlated across items each of the components with *d* and *a*, respectively.

We also plotted the first three components after labeling them according to subject domains (i.e., German, Mathematics) and competence domains (i.e., German reading comprehension, German grammar) to show that the learned embeddings clustered items according to competence and subject domains. To also demonstrate this quantitatively, we computed the average Euclidean distance between the embeddings of items belonging to different competence domains $d_{cd}$ and separately for items belonging to the same competence domain $d_{cs}$. Then we calculated the relative distance difference $RDD_{c} = 100\cdot \frac{d_{cd}-d_{cs}}{d_{cs}}$. To appraise whether the observed $RDD_{c}$ was significant, we compared it with the null distribution obtained by permuting the competence domain labels 1,000 times. We also computed the average Euclidean distance between the embeddings of items belonging to different competence domains but the same subject domain on the one hand, $d_{ss}$, and for items belonging to different competence domains and also different subject domains, $d_{sd}$ (this ensured that the similarity between item embeddings of the same subject domain was not driven by the similarity between items of the same competence domain, shown above). As before, we computed the observed relative distance difference $RDD_{s} = 100\cdot \frac{d_{sd}-d_{ss}}{d_{ss}}$ and compared it with the corresponding null distribution obtained by permuting the competence domain labels 1,000 times.

To further ascertain whether the learned item embeddings reflected the structure of the curriculum, for each competence domain we computed two cluster validity indices with respect to the labels defined by the different competences. These indices measure the similarity of the embeddings of items of the same cluster (here, a particular competence) relative to those from other clusters. The first cluster validity index was the Calinski-Harabasz index [46], which measures the ratio of the sum of between-cluster dispersion (distances between the cluster centroids and the global centroid) and of within-cluster dispersion (distances between embeddings within a cluster and its centroids). The second cluster validity index was the inverse of the Davies-Bouldin index [47]. The DB index is defined as the average similarity (i.e., within-cluster distances to between-cluster distances) of each cluster with its most similar cluster. The Davies-Bouldin index is thus a more strict measure of clustering as it assumes the worst-case scenario. This index was inverted because the DB index, as opposed to the Calinski-Harabasz index, is defined such that lower values denote more well-defined clusters. To ensure that the observed validity index values were not due to chance, we computed the null distribution of the cluster validity indices by permuting the assignment of competences to items 1,000 times.

Finally, to discard that the similarities between embeddings reflected only the fact that items corresponding to different competences had different levels of difficulty (as measured by an IRT model), we split the items within each competence domain in 10 levels (given that there were 7 different grades, we chose this number so that it would result in more than one level of difficulty per grade, guaranteeing that the items within each subset had a very similar difficulty level). We then computed the cluster validity indices for each difficulty level. After slicing the data in this way, some of the resulting subsets had very few items, precluding robust statistical analyses. We therefore focused the analysis on those subsets with at least 10 competences and at least 30 items per competence. Similarly as before, we constructed the null distribution of the cluster validity indices by permutation. To control for the multiple tests corresponding to the different levels of difficulty within each competence domain, we constructed the null distribution of the maximum statistic across difficulty levels. This analysis was done separately for each competence domain.

## Figures and Tables

**Fig. 6:**
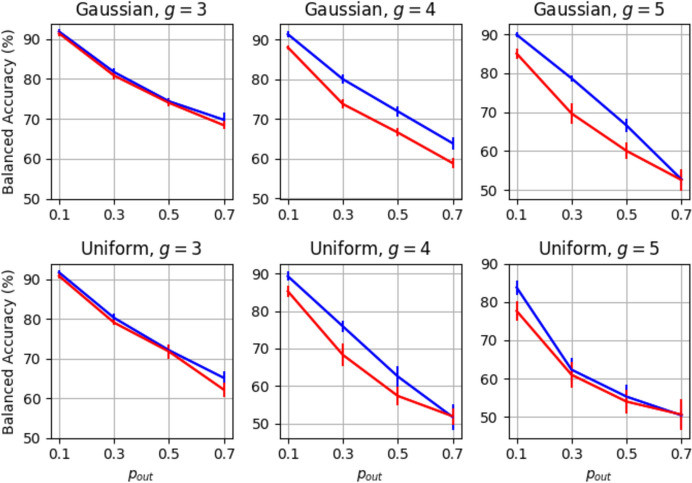
Comparison between the balanced accuracy scores obtained by the GNN (blue) and IRT (red) models on synthetically generated data. The rows represent the results for Gaussian (top) and uniform (bottom) degree distributions, while the columns show data for three, four, and five ability groups. The x-axis represents the probability $p_{out}$ that the students answer correctly items from the group for which they are not proficient, and the y-axis the balanced accuracy. Error bars denote the standard deviation across 10 runs, using 10-fold cross-validation. $p_{in}$ was fixed to 0.7

## Data Availability

The code used for the analysis of the ability estimates and generation of the reported results is available on Github: https://github.com/lisiq/Modelling-Students-Learning. The data set used in this study is not publicly available because Northwestern Switzerland’s four cantonal authorities (i.e., the contracting authorities) own it. Requests to access the data set should be directed to the main office of the four cantons (kommunikation@bildungsraum-nw.ch).

## Appendix A GNN architecture

Our model follows an Encoder-Decoder architecture Hamilton et al. [48]. The Encoder consists of two GraphSAGE layers Hamilton et al. [49], a state-of-the-art technique for inductive representation learning on a large-scale graph, with an Exponential Linear Unit (ELU) nonlinearity in-between.

Mother tongue and gender were entered as student features, competence domain (one-hot encoded) as item feature, and age and grade at which the item was responded as edge features. Student and item features were linearly transformed and added to respective embedding layers, which provided additional parameters. The result was then fed into the first GraphSAGE layer.

The embeddings learned by the Encoder are used by the Decoder (classifier) to make predictions via an output linear layer, where the input (edge embedding) of the Decoder is the concatenation of a source and target node (i.e., student and item) embeddings and the initial edge features.

## Appendix B implementation

In order to manage the computational complexity associated with large graph data, we employed the NeighborLoader method. This technique partitions the graph into manageable batches, each of size $2^{16}$. The partitioning process is guided by a sampling strategy that prioritizes the immediate neighborhood of each node. Specifically, for each node, we sampled all immediate neighbors, followed by all neighbors-of-neighbors. This hierarchical sampling strategy ensures that the most relevant local information is captured for each node in the batch. Furthermore, it aligns with our choice of GraphSAGE as the primary algorithm for representation learning. GraphSAGE has been empirically demonstrated to excel in learning representations on large graphs, making it an ideal fit for our data structure and sampling strategy (Table 1).

**Table 1:** An overview of the GNN model architecture and the chosen hyperparameters

Layer		Input dimension	Output dimension	Activation function
Encoder	Embedding students	1	12	–
	Embedding items	1	12	–
	Linear layer students	2	12	–
	Linear layer items	11	12	–
	SAGEConv 1	12	12	ELU
	SAGEConv 2	12	12	ELU
Decoder	Linear layer	26	12	ELU
	Linear layer	12	1	–

We employ Adam as the optimizer with a learning rate of 0.005 and Binary Cross Entropy as the loss function. We assess the model’s performance using a 10-fold cross-validation technique and report the average test results of the model that performs best on the validation set. If there is no improvement in the best validation results after 200 epochs, we proceed to the next fold.

To identify the optimal hyperparameters for the GNN model, we conducted a grid search in the hyperparameter space defining the dimensionality of the GraphSAGE layers and of the second linear layer of the Decoder. We evaluate our model’s performance based on the balanced accuracy and AUC.

## Appendix C item response theory model

Besides the GNN model, we also fitted a two-parameter logistic (2PL) Item Response Theory (IRT; Reise et al.; Embretson and Reise [14, 16]) model to the data, to compare their performance and parameters. According to this model, the probability that the response $u_{ij}$ of student *i* to item *j* is correct is given by:

C1 $$\begin{aligned} p(u_{ij} = 1) = \frac{e^{a_j (\theta _i - d_j)} }{1 + e^{a_j (\theta _i - d_j)}} \end{aligned}$$

In this expression, $a_j$ and $d_j$ denote, respectively, the difficulty and the discrimination of the item, and $\theta _i$ the ability of the student. For parameter optimization, we used the same strategy as for the GNN model.

In the MINDSTEPS data set, the IRT model performed equivalently (AUC=0.82 and balanced accuracy=0.74) to the GNN model. In the next section, we present simulations showing conditions in which the GNN model outperforms the IRT model.

## Appendix D synthetic evaluation

We generated synthetic response data with tailored topological patterns in the interactions between students and items so that we could evaluate the performance of the GNN and the IRT models in different settings.

### Synthetic data generation

The data generation algorithm consists of two main components. In the first part, we generate the topology of interactions between students and items. This involves creating a bipartite graph denoted as $G=(V,E)$, where *E* represents the set of interactions between the node sets $V_s$ (students) and $V_i$ (items). In the second part, we assign response labels to the student-item interactions. Each interaction $e \in E$ is assigned a label $l \in {0,1}$, indicating a specific response outcome.

#### Topology generation

We generate the topology using the Stochastic Block Model (SBM; Karrer and Newman [50]). Originally introduced to create graphs with clusters of closely connected nodes, the SBM has evolved to accommodate various features such as degree distributions and more. This flexibility makes the SBM a valuable tool for creating graphs with both predetermined characteristics and stochastic elements. While the SBM is commonly used to generate unipartite graphs with *g* groups, it can be easily extended to generate bipartite graphs by defining 2*g* groups and ensuring that connections between nodes in the same partition are impossible. In our case, this translates to forbidding student-student and item-item interactions. Then, for example, a bipartite community structure can be encoded by making students belonging to group $g_s$ more likely to interact with items from group $g_i$ if $g_s=g_i$. In our experiments, we used the *Degree-Corrected* Stochastic Block Model (DC-SBM), which allowed us to encode both the group structure and the expected degree distribution within the generated graph. The DC-SBM generates more realistic graphs than the vanilla SBM, as nodes within real-world graphs typically show degree heterogeneity even if they are part of the same community.

The data-generation algorithm takes the following inputs: the number of students $n_s$, the number of items $n_i$, a partition of the nodes into 2*g* groups, and a square matrix $B \in \mathbb {R}^{2g \times 2g}$ where each element $B_{jk}$ defines the group structure of the graph. Additionally, since we use a DC-SBM, we provide a vector $\varvec{\theta } \in \mathbb {R}^{n_s + n_i}$, where each element represents the expected degree of the corresponding node. Based on these parameters, we independently connect nodes *u* and *v* with probability: $P((u,v)) = \frac{B_{g_u,g_v} \theta _u \theta _v}{2m}$ where $m=\sum _{j \in V_s \cup V_e} \theta _j$, and $g_u$ and $g_v$ indicate the groups of nodes *u* and *v* respectively. The $B_{g_u, g_v}$ matrix, which encodes the bipartite structure of the student-item relationships, is defined as follows: $B_{g_u, g_v} = 1$ if $g_u = g_v$ (i.e., student/item *u* item/student *v* are in the same group), and $\frac{1}{g - 1}$ otherwise (where *g* is the total number of groups).

#### Label generation

We determine a student’s probability of answering an item correctly based on whether the group $g_i$ of the item matches the group $g_s$ of the student. For each edge $e = (s,i) \in E$ in the topology, we assign student *s* a probability $p_{in}$ of responding correctly to an item if $g_s = g_i$. If the groups do not match ($g_s \ne g_i$), we assign a probability $p_{out}$ for the student to answer the item correctly, where $p_{out} < p_{in}$. If a student correctly answers an item, the edge *e* is labeled $l=1$; otherwise, the edge is labeled $l=0$.

### Synthetic data results

In this section, we report the performance of the GNN and the IRT models on the synthetically generated data described above.

We simulated graphs with $n_i + n_s = 1000$ nodes in which we vary the number of groups $g \in \{3,4,5\}$. We fixed $p_{in}$ to 0.7, and varied $p_{out}$. We used both a relatively homogeneous *Gaussian* degree distribution and a more heterogeneous *uniform* one to ensure we covered different scenarios. To fix the degree distribution, for the Gaussian case, we sampled the expected degree vector $\varvec{\theta } \in \mathbb {R}^{n_s + n_i}$ from a Gaussian distribution with mean $\mu = \frac{n_i+n_s}{g}$ and standard deviation $\sigma = \frac{n_s + n_i}{100}$. For the uniform case, we sampled $\varvec{\theta }$ from a discrete uniform distribution over the range $\left[ 1, \left\lfloor \frac{n_s + n_i}{g} \right\rfloor \right]$, where the (inner) square brackets indicate that we are taking the integer division.

We present the balanced accuracy results for different number of groups and degree distributions in Fig. 6. For low values of $p_{out}$ (when students are considerably worse at correctly answering items in the group for which they have not been trained), the simulations show that the GNN tends to outperform the IRT model. As $p_{out}$ increases and approaches $p_{in}$, the performance of both models deteriorates and becomes similar.

## Appendix E conceptual connections between perceptron models, IRT, and GNNs

This section explores the conceptual connections between the perceptron, IRT, and GNNs. All three frameworks share a fundamental similarity in their mathematical formulation. First, the perceptron has the same mathematical expression as logistic regression. It expresses the probability of a binary outcome as $p(u_{ij}=1) = \frac{1}{1+e^{-\beta \cdot \pmb {X}}}$, applying the sigmoid function to a linear combination of features $\pmb {X}$. The 2PL IRT model can be seen as a particular case of the logistic regression framework for psychometric contexts, expressing student response probability as $p(u_{ij}=1) = \frac{1}{1+e^{-a_j(\theta _i-d_j)}}$, where $a_j$ represents item discrimination, $d_j$ represents item difficulty, and $\theta _i$ represents student ability. The main advantage of the IRT model is the domain-informed interpretability of its parameters, facilitating communication with education practitioners. The GNN model for learning the school curriculum considered in our work learns representations for a student item interaction as $[h_s, h_i, f]$ where $h_s$ is the student embedding, $h_i$ the item embedding, and *f* additional edge features (concatenated, as discussed in section A). The representations pass through two perceptron layers, as the perceptron is a fundamental component of all standard neural classifiers.

## Footnotes

1 Grade 3 corresponds to the 5th year of mandatory schooling in the Swiss school system and to the age of 8 years.
